# Investigation of Risk Factors Associated with the African Swine Fever Outbreaks in the Nizhny Novgorod Region of Russia, 2011–2022

**DOI:** 10.1155/2023/6334935

**Published:** 2023-09-21

**Authors:** Olga I. Zakharova, Andrey A. Blokhin, Ivan V. Yashin, Olga A. Burova, Denis V. Kolbasov, Fedor I. Korennoy

**Affiliations:** ^1^Federal Research Center for Virology and Microbiology, Branch in Nizhny Novgorod, Nizhny Novgorod, Russia; ^2^Federal Research Center for Virology and Microbiology (FRCVIM), Volginsky, Russia; ^3^Federal Center for Animal Health (FGBI ARRIAH), Vladimir, Russia

## Abstract

African swine fever (ASF) is a transboundary viral disease that affects both domestic pigs and wild boar, causing enormous damage to the pig production. Numerous studies are devoted to elucidating the role of domestic and wild pigs in ASF outbreaks. The identification of the risk factors contributing to the spread of disease in previously unaffected regions is of special interest. We conducted a retrospective analysis of sporadic ASF outbreaks in the Nizhny Novgorod region of the Russian Federation from 2011 to 2022 among both domestic pig and wild boar populations. Methods of spatiotemporal cluster analysis, mathematical modeling with logistic and negative binomial regressions, as well as the cross-correlation analysis of time series, were used to identify the most significant factors associated with the ASF outbreaks' presence and intensity. Regression analysis did not reveal a statistically significant correlation between ASF outbreaks in wild boar and their population density over the entire study period. However, the spatial and temporal coincidence between outbreaks and increased wild boar density was observed at the beginning of the ASF epidemic in the region. We identified the significant environmental and sociodemographic factors contributing to the spread of ASF in both domestic pig and wild boar populations. The number of small-scale farms (backyards) and summary road length were determined as the main factors contributing to the spread of ASF among domestic pigs in the Nizhny Novgorod region. The regression model for ASF outbreaks in the wild boar population revealed the presence of water bodies, number of hunting farms, and the occurrence of ASF outbreaks in the domestic pig population to be the main explanatory factors. A statistically significant coincidence was identified between the monthly volume of pork products imported to the region from the rest of Russia and the occurrence of ASF outbreaks in both domestic and wild populations. Thus, the identified spatiotemporal patterns may be useful to determine areas with an elevated risk of ASF outbreaks emergence for better resource management, disease control, and prevention. The importance of intensified monitoring and control measures in the pig products supply chain is strongly needed. The obtained models can be applied not only by the veterinary service of the Nizhny Novgorod region but to other regions with similar environmental and sociodemographic conditions.

## 1. Introduction

African swine fever (ASF) is a lethal viral disease of domestic pigs and wild boar, characterized by a superacute, acute, subacute, less often chronic form with a high-mortality rate of up to 100%. The virus belongs to the family Asfarviridae, genus *Asfivirus* [1–3].

The first ASF outbreak in Russia was probably caused by the virus spread via wild boar from Georgia in 2007 [4]. Migration and movement of wild boar and low levels of biosecurity in pig farms remain the main causes of extensive ASF spread in Europe and Asia, including Russia [5–7]. The ASF outbreaks in wild boar and domestic pigs currently continue to emerge in the Russian Federation and particularly in the Nizhny Novgorod region. Despite the implementation of various eradication and surveillance strategies, ASF spreads extensively, demonstrating both endemic and sporadic patterns [8].

The lack of information on ASF spreading from wild boar to domestic pigs and vice versa increases interest in this problem. Recent studies have shown that the movement of infected animals, importation of contaminated pork products, and hunting activity are significant factors in the transmission of ASF between domestic pigs and wild boar populations [9–11]. Studies of the local ASF outbreaks in some European countries were aimed at testing the density of wild boar populations as a significant factor determining the spread of the disease [12–15]. In some cases, the deaths of wild boar from ASF were registered near farms [4, 16]. Spatiotemporal analysis in the Russian Federation showed that ASF outbreaks in both domestic pigs and wild boars correlated to each other [17, 18]. The spread of ASF in European countries demonstrated the significance of the wild boar population density factor, although the wild boar was not the original cause of the infection. Numerous infected wild boar carcasses were found near national borders, such as in Russia near Georgia, and in Poland and Lithuania near the borders of Belarus and Ukraine, suggesting wild boars to be a remarkable factor of the ASF virus transboundary transmission [4, 19].

The use of intensive methods for wild boar population control such as depopulation is possibly one of the major causes of ASF spread in certain regions of Russia. Depopulation strategy may result in the migration of infected animals and the formation of new fomites in the environment [10, 16].

However, the ability of the ASF virus to persist at consistently high levels in wild boar populations is still uncertain [20, 21]. Spatiotemporal analysis of ASF spread in the Russian Federation between 2007 and 2012 showed that outbreaks were registered both in close proximity to each other and at hundreds kilometers apart. Apparently, it cannot be associated only with the migration of wild boar populations [22–25]. The time intervals between the detection of ASF cases can range from zero to several weeks. The spatial and temporal dynamics of ASF spread in wild boar are influenced by a multiplicity of factors, including physical, geographical, and socioeconomic ones. For instance, the geographical distribution of domestic and wild boar populations and the frequency of their interactions determine the spread of ASF, as well as the density of road networks and water bodies [26–28].

The presence of ASF-infected wild boar carcasses in the wild nature supports the ASF virus persistence even at low-wild boar population density, especially given the virus stability in the wide range of environmental conditions. [29].

Pepin et al. [30] demonstrated that 53%–66% of ASF cases in wild boar were caused by direct or indirect contact with contaminated carcasses of animals died from ASF. Gervasi and Guberti [21] showed that the ASF virus persistence in the environment is mainly maintained through the contaminated wild boar carcasses, which can act as the virus reservoir in ASF foci, until the population density recovers and achieves the level enabling an infection transmission via direct effective contact.

The emergence of ASF outbreaks may be related to the movement and importation of contaminated pig products or livestock [31]. However, full data on the movement of agricultural products and livestock are rarely available to researchers.

**Figure 1:**
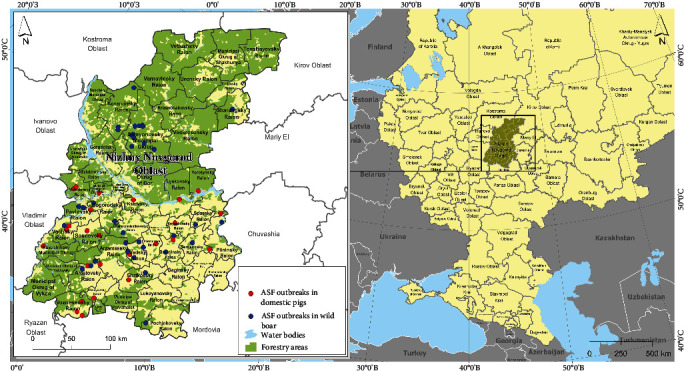
ASF epidemic situation in the Nizhny Novgorod oblast from 2011 to 2022.

The Nizhny Novgorod region of Russia has been affected by the ASF outbreaks since 2011 (Figure 1). The first ASF outbreak in the region was recorded in a backyard in the spring of 2011, involving a single case in domestic pigs. The subsequent ASF outbreaks started to occur sporadically from October 2016, causing significant losses for hunting and domestic pig farms. The highest number of ASF outbreaks among the wild boar population (20) was registered in the Semenovsky district in 2017. Other southern districts such as Koverninsky, Pavlovsky, and Bogorodsky also reported single outbreaks. From 2018 to 2021 ASF outbreaks among wild boar occurred annually in the same areas. Domestic pig livestock in the Nizhny Novgorod region is mainly present on smallholder farms, forming a broad network. ASF outbreaks were registered in small farms to a lesser extent than in wildlife. The spread of ASF in domestic pig populations was primarily observed in the southern areas of the region, with denser locations of smallholder farms. To control the outbreaks and prevent further spread, it is crucial to study the dynamics of the ASF outbreaks in both wild boar and domestic pig populations and identify any correlations with key risk factors. Hence, our research purpose was an epidemiological analysis of ASF outbreaks in domestic pigs and wild boar populations in the districts of the Nizhny Novgorod region as well as identification of the spatial and temporal distribution of the disease and revealing the leading risk factors. We also analyzed data on the transportation of pork products and livestock into the Nizhny Novgorod region from other regions of Russia and examined their correlation with the intensity/presence of ASF outbreaks.

## 2. Material and Methods

### 2.1. Study Area

The Nizhny Novgorod oblast (or region) is a first-level administrative division of the Russian Federation that is located in its European part (Figure 1). The region is divided into 52 s-level divisions (districts), which were the model units for our study being the minimum units of availability for some data. The Nizhny Novgorod oblast occupies an area of 76.9 thousand km^2^ or 0.31% of the whole Russia. It stretches over 430 km from north to south along the 45th meridian and about 300 km from West to East at its widest part. The climate is characterized as temperate continental. The average population density is around 40 people per km^2^. The region has a vast river system, with a total length of about 32,000 km. The Nizhny Novgorod oblast is covered with over 3,415 thousand hectares of forest, representing 53% of the entire territory. In the northern part of the region, the proportion of forests in the total area reaches almost 80%.

Agriculture is quite diverse in the Nizhny Novgorod region. The pig smallholder sector is represented by backyards, with 98.89% of the total pig livestock of the region. The Nizhny Novgorod region has 14 wildlife preserves and reserves, where wild boar hunting is prohibited. The population density of wild boar in the region is decreasing every year due to depopulation measures of the wild boar aimed to prevent ASF outbreaks. As of 2023, the estimated total number of wild boar in the region is 1,326. The highest density of wild boar is observed in the northern and southeastern parts of the region that borders the republics of Chuvashia and Mari-El. The wild boar density ranges from 0.047 to 0.086 animals per km^2^.

### 2.2. Research Outline

Multistage analysis was performed to identify the most significant factors associated with the ASF outbreaks emergence in domestic pigs and wild boars in the Nizhny Novgorod region between 2011 and 2022. It involved the identification of the correlation between the presence/number of ASF outbreaks and several potential explanatory variables, both dynamically changing over time and stationary:

1. Spatiotemporal cluster analysis was used to identify possible spatial and temporal overlap of clusters of increased wild boar population density with clusters of ASF cases in this population.
2. The logistic regression model was applied to determine the potential relationship between the presence of ASF outbreaks in wild boar and dynamically changing wild boar population densities.
3. Logistic and negative binomial regression models were applied for revealing the relationships between ASF-infected districts of the Nizhny Novgorod region and some static factors for the domestic pigs as well as the wild boar population.
4. The correlations were examined between the time series of ASF emergence in the whole region and the intensity of live pigs and pork products' importation from other regions of the country monthly from 2019 to 2021.

### 2.3. ASF Data

According to data collected until September 1, 2022, 66 outbreaks of ASF were registered in the Nizhny Novgorod region. An outbreak is defined as the laboratory-confirmed detection of the ASF virus in one or more wild boar or domestic pigs linked to a geographically defined location such as a single farm, settlement, hunting ground, or in the wild. For each outbreak, information is available on the number of infected (“cases”) and susceptible individuals. ASF outbreak information was obtained from the FGBI Veterinary Center (https://xn----8sbfkcavba6bf4aedue4d.xn--p1ai/o-nas/informatsiya/epizooticheskaya-obstanovka). Laboratory confirmation of ASF was carried out by the ASF reference laboratory of the Federal Centre for Animal Health (FGBI ARRIAH) (https://arriah.ru/ic/laboratorno-diagnosticheskiy-tsentr/struktura/referentnaya-laboratoriya-po-afrikanskoy-chume-sviney/) using standard PCR diagnostic methods.

### 2.4. Wild/Domestic Pig Population Data

The data on wild boar population number by district for each year from 2011 to 2022 were obtained from the Committee for the protection, use, and reproduction of wildlife of Nizhny Novgorod region (https://ohotnadzor.government-nnov.ru/). Due to intensive wild boar depopulation measures, these numbers vary significantly over the study period, so the wild boar density was further considered a dynamically changing variable (Figure S1).

Data on the number, density, and distribution of domestic pig populations were sourced from the official website of the Federal State Statistics Service (https://rosstat.gov.ru/). Information about domestic pig population distribution in personal subsidiary farms and their number was obtained from the Veterinary Committee of the Nizhny Novgorod region (https://vetnadzor.government-nnov.ru/).

### 2.5. Livestock and Pork Products Movement Data

The data on the movement of live pigs and pork products into the Nizhny Novgorod region were obtained from the government information system “Mercury.” This system launched in 2019 is designed for electronic certification and surveillance of cargo subject to state veterinary supervision during production, circulation, and movement throughout the Russian Federation (https://mercury.vetrf.ru/). We obtained two sets of monthly data from 2019 to 2021, namely: (1) the number of live pigs supplied from all regions of the Russian Federation to the Nizhny Novgorod region (heads); (2) the total mass of pork products supplied from all regions of the Russian Federation to the Nizhny Novgorod region (kg). No information was available for our study on the regions from where the pigs/products were supplied.

### 2.6. Environmental and Sociodemographic Factors

Environmental variables were collected from the open street map (OSM) vector and raster GIS layers of the Nizhny Novgorod region (https://www.openstreetmap.org/#map=3/69.62/-74.90). It included the percentage of water bodies (rivers and lakes), the vegetation cover of the area, the length and density of roads etc. All variables were extracted and summarized by district area with the calculation of median values using zonal statistics tools in the ArcGIS Pro software (Esri, Redlands, California, USA).

All variables were selected based on their significance in the occurrence and spread of ASF in wild boar and domestic pigs described elsewhere [32]. All variables were preliminarily analyzed for mutual correlation using the Spearman correlation test with a threshold value *r*_*s*_ = 0.7 to avoid multicollinearity. In each pair of correlated variables, the one that showed the lowest correlation with other variables was left for analysis. The correlation analysis revealed collinearity between several variables, such as the number of hunting farms and the number of feedlots per hunting farm, vegetation cover area including shrub stands and the total number of feedlots per hunting farm in an area, total vegetation cover area and the number of hunting farms in an area, the total number of domestic pigs and stocking densities per rural settlement. Subsequently, a multiple correlation analysis using a variance inflation factor (VIF) test was performed in the R 4.2.1 software environment (R Core Team, 2022) on the remaining pairs of predictors. Those factors demonstrating VIF less than 5 were identified for further regression analysis [33, 34]. All factors included in the analysis are presented in Table 1 and visualized in Figures S2–S4.

### 2.7. Methods of Analysis

#### 2.7.1. Spatiotemporal Cluster Analysis

Spatial–temporal cluster analysis was performed to evaluate the possible overlapping between the increased ASF incidence in wild boar and their population density. Kulldorff moving window scan statistics method was used [35, 36]. This method identifies circular windows in the study area where ASF cases were clustered more densely than expected under the null hypothesis assuming their random distribution. The analysis uses a cylindrical scan window, where the vertical dimension represents time. The model units were the districts of the Nizhny Novgorod region, while the analyzed values were attributed to the centroids of the districts. First, the normal model was used to identify the areas where wild boar density is higher than would be expected under its hypothesized normal distribution. Second, the Bernoulli model was used to identify clusters of increased ASF incidence in the wild boar. Positive data were the number of ASF-infected wild boar in the district and negative were the number of healthy animals. The maximum size of the cylindrical scan window was selected by default to be 50% of the study area and 50% of the study period. The location and time frame of the identified clusters were visually compared to assess if the concentration of ASF cases in wild boar corresponds to the areas with increased wild boar density.

#### 2.7.2. Identification of a Potential Correlation between the Occurrence of ASF Cases and Dynamically Changing Wild Boar Population Densities

Generalized logistic regression analysis was applied to test the hypothesis that the occurrence of ASF cases in wild boar is associated with increased wild boar population density. The logistic regression model predicts the probability of event *Y* occurrence if certain values of factors *X*_*i*_ are realized. The logistic regression is interpreted in terms of odds (odds ratio, OR). The odds of an event is the ratio of the probability of the event to the probability of no event [37].

The response variable (*Y*) used in this regression analysis was categorical, indicating the presence or absence of ASF outbreaks in wild boar population in a district in the respective year. The independent variable was the wild boar population density for the area/time period. The statistical metrics to assess the quality of the model fit were: the Hosmer–Lemeshow test, which assesses whether the observed frequency of events corresponds to that expected in the subgroups of the model population; the Shapiro–Wilk test for normality of regression residuals; the Local Moran's I autocorrelation test to examine the regression residuals for clustering [38].

#### 2.7.3. Identification of the Correlation between ASF-Infected Districts of Nizhny Novgorod Region and a Number of Static Factors for the Domestic Pigs and the Wild Boar Populations

Regression models were used to investigate the relationships between the cumulative intensity (or presence) of ASF outbreaks in each district and various static factors. We tested two models, namely: a logistic model, with the response variable being a dichotomous presence/absence of outbreaks in the area; and a negative binomial model, with the count type response variable being the number of ASF outbreaks. Multiple explanatory factors listed in Table 1 were included in both models.

Negative binomial regression model is a certain type of regression applied to count data when the variation of the response variable exceeds its mean value (i.e., when overdispersion is observed) [39]. The choice of negative binomial regression in our case was justified by the distribution of the number of outbreaks in both wild boar and domestic pigs, which demonstrates a pronounced over dispersion. For ASF outbreaks in domestic pigs the mean is 0.49, variation is 95.45; for outbreaks in wild boar, the mean is 0.74, while variation is 32.41.

Regression models were adjusted using stepwise removal of independent variables to achieve the lowest Akaike information criterion (AIC) using the stepAIC procedure. The significance of the variables was assessed using the Student *t*-test with a corresponding *p*-value. The overall quality of the models' fit was assessed with adjusted R2, representing the share of variation accounted for by the model.

#### 2.7.4. Identification of the Correlation between the ASF Outbreaks in the Region and the Intensity of Live Pigs and Pork Products Importation from Other Regions

Logistic regression was used to assess the correlation between the monthly occurrence of outbreaks in both populations and the imports of pork products and live animals. The presence or absence of ASF outbreaks in wild boar and domestic pigs for each month for the region was considered as a response variable, and data on the total volume of pork products and the number of live pigs imported to the region from other regions of the country were used as independent variables. Additionally, the time series of ASF outbreaks in domestic pig and wild boar populations were tested for cross-correlation with the time series of live animal and pork product importation. Cross-correlation is a measure of the similarity of two-time series as a function of the bias of one event relative to the other [40, 41]. Such an analysis enables evaluating both the similarity of time series (on a scale from −1 to 1) and the temporal shift of one series to another.

### 2.8. Software

Open-source statistically oriented programing environment R 4.1.0 (R Core Team, 2021) was used to test variables and perform regression analyses. The R software packages “car” and “plyr” were used to estimate the variance inflation factor (VIF) [42, 43]. All predictor variables with a VIF threshold greater than 5 were excluded from the analysis [44]. The R “MASS” software package was used to fit the regressions [45]. The stepAIC function was utilized step by step to exclude variables when fitting regression models. Time series cross-correlation was evaluated with a ccf function [46]. Spatial analysis and data visualization were performed using geographic information systems ArcGIS Desktop 10.8.2 and ArcGIS Pro 3.0 (Redlands, CA, USA).

## 3. Results

### 3.1. Epidemiological Analysis

Twenty-six (26) ASF outbreaks in domestic pigs and 40 in wild boar were registered in the Nizhny Novgorod region during the study period (Figure 1). The number of susceptible domestic pigs in outbreaks ranged from 1 to 274 (median 9), while the number of infected pigs ranged from 1 to 47 (median 2). The number of cases in wild boar outbreaks ranged from 1 to 35 (median 1).

**Figure 2:**
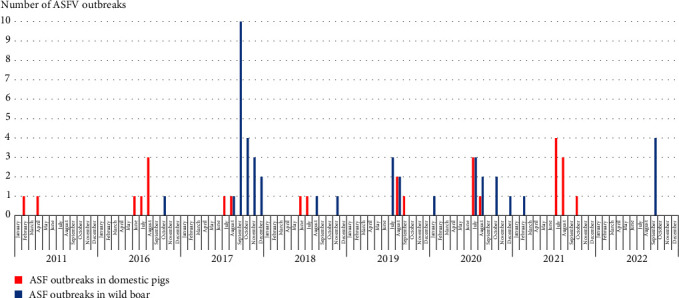
Diagram of the seasonality of ASF in wild boar and domestic pigs' populations in the Nizhny Novgorod region for the 2011–2022.

The temporal diagram (Figure 2) shows the obvious presence of seasonality in ASF emergence in the region. ASF outbreaks in wild boars mostly occurred from midsummer to late autumn, which coincides with the hunting season from June 1 to February 28 (29).

### 3.2. Spatiotemporal Cluster Analysis

**Figure 3:**
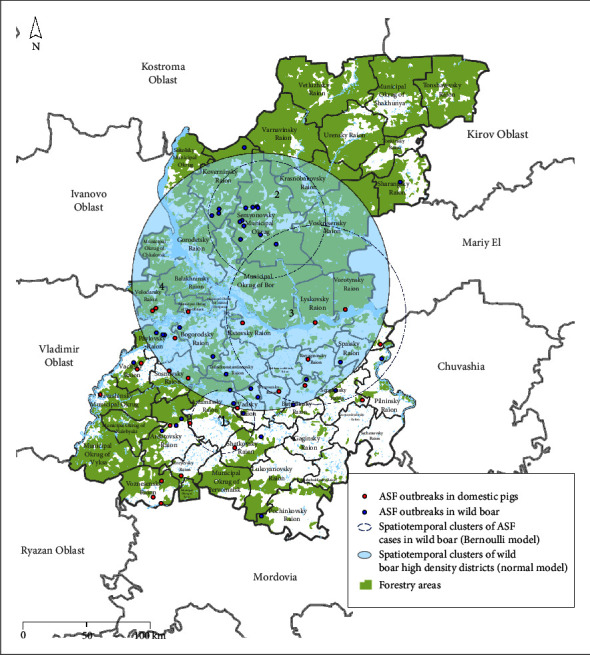
Spatial–temporal clusters of ASF outbreaks and areas of high-wild boar population density in the Nizhny Novgorod region, 2011–2022 (yearly distribution of wild boar density is presented in Figure S1).

A cluster of ASF incidence in wild boar overlapping with cluster of increased wild boar population density was found in the central part of the study area in 2016 (Figure 3, Table 2).

### 3.3. Correlation of ASF Outbreaks with Wild Boar Population Density (Logistic Regression Results)

The logistic regression model demonstrated a failure of wild boar population density as an explanatory variable for the presence of ASF outbreaks in this population (*p*-value = 0.54 > 0.05). Analysis of wild boar density in dynamics for 2016–2022 showed that at the beginning of the ASF history in the region, ASF in wild boar occurred mainly in the northern part of the region, where wild boar population density ranged from 0.025 to 0.068 animals per km^2^. In subsequent years, ASF outbreaks in wild boar shifted to the southern districts of the study region, where wild boar density was much lower than 0.025 wild boar per km^2^ (Figure S1).

### 3.4. Regression Analysis of Risk Factors for ASF Outbreaks in Domestic Pig and Wild Boar Populations

The obtained statistical metrics for different regression models are shown in Table 3. It can be seen that the correlation for outbreaks among domestic pigs is better explained by the logistic model, and by the negative binomial model for outbreaks among wild boars.

The results of regression analysis of registered ASF cases among wild boar with a number of climatic and sociodemographic factors using negative binomial models are presented in Table 4.

**Figure 4:**
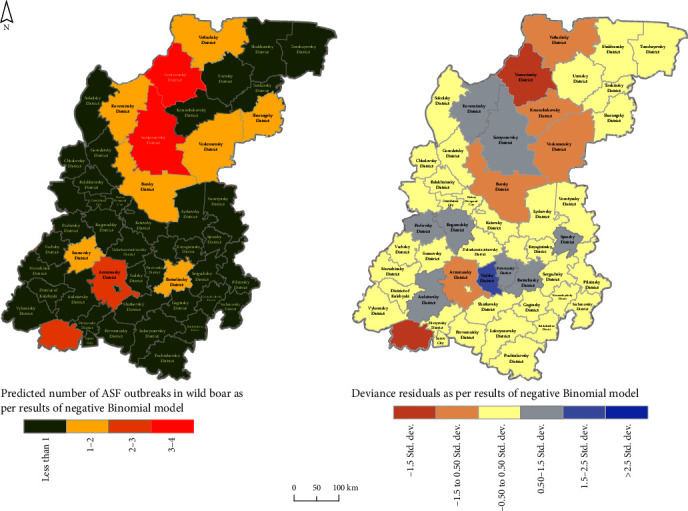
The predicted number of ASF outbreaks in wild boar in the Nizhny Novgorod region as estimated by the negative binomial regression model, and the distribution of the associated regression residuals.

The negative binomial regression model predicted the different number of outbreaks in the wild boar population in the Nizhny Novgorod regions. From 3 to 4 outbreaks were predicted for Semenovsky and Varnavinsky districts, 2–3 for Arzamassky and Voznesensky districts, and 1–2 outbreaks in southern districts like Sosnovsky and Buturlinsky, and also for the northern part of the region: on the northeastern side are Borsky, Voskresensky, and Sharangsky districts bordering the Kirov region and the Republic of Mari-El, and on the northwestern side are Vetluzhsky, Koverninsky districts bordering the Kostroma region (Figure 4, left). These districts have well-developed hunting farms and rich vegetation cover in the form of coniferous and broad-leaved forests. This corresponds well with the obtained results of the regression model on the main risk factors. The significant predictors of the registered number of ASF outbreaks among wild boar in the Nizhny Novgorod region are the share of surface water bodies in the area, the number of hunting farms, and the presence of outbreaks among domestic pigs in the area. The model residuals showed an almost random distribution, indicating a good fit (Figure 4, right).

**Figure 5:**
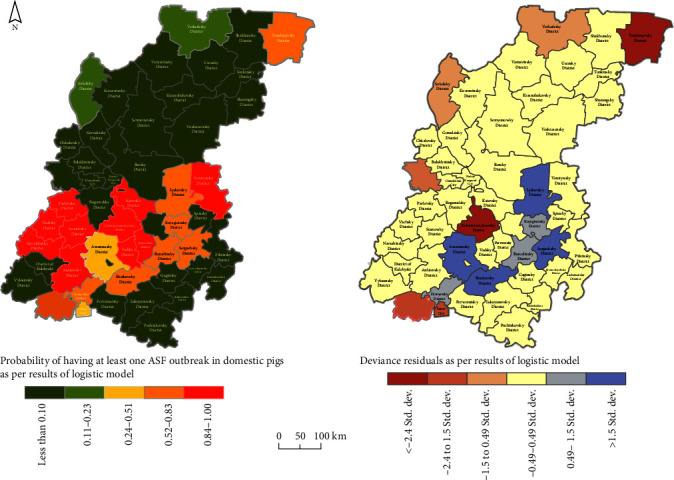
The predicted probability of emerging ASF outbreaks in domestic pigs and the distribution of the associated model residuals in Nizhny Novgorod region estimated by the logistic regression.

Table 5 presents the results of modeling the correlation of ASF outbreaks among domestic pigs with several factors using a logistic regression model. Regression analysis of the correlation between the occurrence of ASF in domestic pigs found a correlation primarily between the number of private farms and the length of roads. The coefficient of determination was 0.70.  Figure 5 shows a map of the model distribution of the probability of outbreaks (left) of ASF among domestic pigs, as well as the distribution of regression residuals (right).

The logistic regression model predicted that the districts of Nizhny Novgorod region located in the southwestern and southeastern parts of the region are most susceptible to ASF emergence in domestic pigs (Figure 5, left). The highest probability of ASF outbreaks is predicted in Pavlovsky, Vachsky, Navashinsky, and Sosnovsky districts, as well as in the south–eastern part of the region—Vorotynsky district, where the highest number of smallholder farms involved in breeding and keeping domestic pigs is observed. The model residuals were also distributed without clustering randomly (Figure 5, right).

### 3.5. Revealing Relationships between the Occurrence of ASF Outbreaks and the Volume of Live Pigs and Pork Products Importation to the Region

Logistic and negative binomial models were used to test for potential relationships between the monthly occurrence of ASF outbreaks and the volume of live pigs/pork products importation to the Nizhny Novgorod oblast from the rest of the regions of Russia for 2019–2021. The volume of pork products' importation was found to be a significant predictor (*p* < 0.05), while neither model had enough predictive power with the number of imported live pigs as an explanatory variable (*p* > 0.05). The negative Binomial model performed better for both domestic pigs and wild boar outbreaks (Table 6).

**Figure 6:**
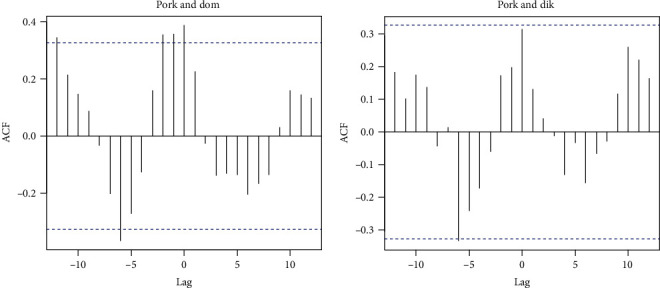
Cross-correlation plot between pork products import and: ASF outbreaks in a domestic pigs population (left) and ASF outbreaks in wild boar (right).

A cross-correlation of time series analysis was performed between the monthly sequence of ASF outbreaks in domestic/wild pigs and pork production supplies, as shown in Figure 6. It demonstrated a correlation between production supplies and outbreak registration. For domestic pigs, the maximum autocorrelation function (ACF) corresponds to a correlation coefficient of 0.32, with the peak of product supply being 0–2 months ahead of the peak of ASF outbreaks (Figure 6, left). A similar, although statistically insignificant correlation is observed for the wild population (Figure 6, right).

## 4. Discussion

### 4.1. Seasonality of ASF Outbreaks in Wild Boar Population

To stop the further spread of ASF in the Nizhny Novgorod region, it is essential to comprehend the biological connections involved in the outbreak chain and identify the primary modes of infection expansion. Nevertheless, the risk factors identified in the literature outline the primary areas of concern and often display distinct features based on the geographic location [30, 47, 48]. Figure 2 shows that ASF outbreaks in domestic pigs were mainly registered in spring and summer, possibly due to intensive movement and importation of infected live animals or products from other areas, and low biosecurity of livestock care. ASF outbreaks in the wild boar population in the region were observed in the late summer, fall and winter months, ending the hunting season. This information gives reasons to speculate that ASF cases could be associated with movements or autumn migrations of wild boar during the rutting period, as well as possible hunter activities during the hunting season. Mentioned above was connected with social regroupings of animals and preparation for the winter season (concentration of local groups). It should be mentioned that the northern districts of the study region present large forestry areas with relatively high number of hunting farms, thus introducing a potential sampling bias (i.e. providing better conditions for regular monitoring and discovering infected animals/carcasses).

### 4.2. Assessment of ASF Spread Risk Factors in Wild Boar Population

The analysis of spatial and temporal patterns of ASF outbreaks in wild boar in the Nizhny Novgorod region from 2016 to 2022 identified areas of elevated incidence in wild boar (Figure 3). Registration of single ASF outbreaks in wild boar in the districts of Nizhny Novgorod region, such as Ardatovsky, Arzamassky, Vadsky, Buturlinsky, Sergachsky, Shatkovsky, and Knyagininsky, may indicate the formation of stationary and localized outbreaks as a result of the persistence of the infection. Spatiotemporal patterns of ASF outbreaks in the districts of Nizhny Novgorod region identified by the analysis allow us to assume that the possibility of the virus transmission still exists even with low wild boar density (0.009 … 0.025 animals per km^2^ of hunting area), which corresponds to the findings in [49]. According to the Action Plan to prevent the introduction and spread of ASF in the territory of the Russian Federation [50], the recommended wild boar density for Nizhny Novgorod Region should not exceed 0.025 animals per km^2^. In some areas, the actual density was much lower than the threshold (Figure S1). Despite measures on wild boar depopulation following the Action Plan, local ASF outbreaks in wild boar continue to be registered in the previously ASF-infected areas of the region. The results of the analysis indicate the possible presence of a common natural reservoir of infection—an environment contaminated with the ASF virus [11, 51–53]. Regression analysis of the ASF outbreak intensity and the density of the wild boar population of the Nizhny Novgorod districts did not detect a statistically significant pattern.

Environmental factors directly related to ASF, describe spatial and temporal patterns of ASF occurrence. It is known that the condition of forest cover potentially influences the preservation of suitable habitat conditions for wild boar. The presence of water bodies is another key predictor of ASF occurrence in the districts of Nizhny Novgorod region.

The regression model result indicating the percentage of water bodies as one of the main predictors correlates with the results obtained in the Europe countries. The mentioned study examined the correlation of ASF-affected regions with surface water by means of the forest-based classification and regression model. A reliable association was found between the detection of at least one case of ASF in wild boar with the proportion of inland wetlands (inland bogs and peatlands) and water bodies, which included watercourses, ponds, and coastal lagoons [54–56].

The ASF spread among wild boar is influenced by ASF outbreaks in domestic pigs. As hypothesized by Guinat et al. [16], insufficient biosecurity measures during hunting and potential direct contact between wild boar and improperly utilized contaminated remains or domestic pigs dying from ASF or contaminated fomites left in a field around small-scale pig farms, may stipulate the ASF virus transmission from domestic to wild populations.

ASF emergence in either wild boars or domestic pigs can put nearby populations at risk of being exposed to the ASF virus. This means that the closer an ASF outbreak is, the higher the probability of susceptible animals developing ASF, which is important in animal disease control. Understanding the time and distance between previous cases of ASF can also help distinguish different epidemiological scenarios of how the virus could invade the resistant populations of wild boar [57, 58].

### 4.3. Assessment of ASF Spread Risk Factors in Domestic Pigs Population

According to the results of a literature review, the most frequently cited factors that significantly influence the occurrence of ASF outbreaks in domestic pigs were related to domestic pigs, often kept in smallholder farms or backyards for owners' use [26]. The study also found correlations between risk factors for new outbreaks of ASF in domestic animals, including the density of pig populations, adherence to biosecurity measures, socioeconomic factors, and population numbers [59, 60]. The more intensively developed the road infrastructure between both municipal districts and regions, the more intensive the movement of animals between the ASF-free and the ASF-affected regions due to the illegal trade in live pigs. We observed a negative correlation between ASF outbreaks and the length of the road network. This suggests that ASF outbreaks may predominantly occur in less economically developed areas, further away from urban centers in the region, with a predominance of small farms with low-livestock numbers.

A prior analysis of ASF epidemics in Russia showed a connection between the distribution of ASF cases among domestic pigs, transportation routes, and population density. We proposed a hypothesis that the spread of the ASF epidemic in the Nizhny Novgorod area might have happened due to the trade of pigs and pig products, which was also studied by [61–63]. According to a study by Taylor et al. [64], the legal pig trade was considered a significant risk factor of the ASF virus transmission to the domestic pigs population of Western European countries. Our study showed a significant positive correlation between the supply of pig products to the region and the presence of ASF outbreaks in domestic pigs. This conclusion is evidenced by results previously obtained by Costard et al. [65]. The researchers mentioned above used a semiquantitative mathematical model to address the lack of knowledge on the risk of ASF spreading through the transportation of pig products. Their approach was based on identifying factors that affect the likelihood of infected meat being produced and sold, and how this could lead to new outbreaks of ASF in the domestic pigs. Their results showed that the illegal importation of pork and ASF-contaminated pig products is a significant risk factor for the introduction of the ASF virus into ASF-free countries [65]. Similarly, ASF-contaminated pork products could be the cause of virus transmission to the domestic pig population in the Nizhny Novgorod Region. Meanwhile, the number of ASF outbreaks in domestic pigs is typically 0–2 months behind peak supplies, which may indicate a cause-and-effect correlation. We do not have enough information on how pig products are distributed from different districts in the region, and we also do not have data on the suppliers, which makes it difficult to determine whether the products come from areas affected by the ASF or not. However, the available data support previous research on the spread of ASF through the transportation of agricultural products and suggests that authorities should increase the monitoring and regulation of product movements.

## 5. Conclusions

In the Nizhny Novgorod oblast, cases of ASF in wild boars are typically not affected by the population density of the species, but areas with higher densities tend to see a wider disease spread during initial outbreaks. ASF outbreaks in domestic pigs are usually found in less developed areas with a higher number of smallholder pig farms. The introduction of ASF to a region might be traced back to the import of pork products from the other areas of Russia.

## Figures and Tables

**Table 1:** List of the variables that were extracted or computed for the retrospective analyses of ASF emergence in the Nizhny Novgorod (Russia), 2011–2022.

Variables	Unit	Median (minimum–maximum)	VIF (variance inflation factor)
Percentage of water bodies	%	0.6 (0.07–17.3)	3.4
Swamp area	km^2^	13.7 (0–7,989.3)	1.73
Vegetation area	km^2^	495.5 (3.1–2,937.8)	4.39
Percentage of vegetation area	%	35.7 (035–81.8)	3.23
Number of rural settlements	unit	83.5 (2–1,518)	2.98
Hunting grounds area	km^2^	1,213 (215–8,401.8)	1.76
Number of feeding grounds	unit	9.5 (0–58)	3.91
Number of hunting farms	unit	3 (0–13)	2.71
Summary road length	km	543.7 (21.1–1,410.0)	2.45
Road density	km^2^	0.42 (0.29–0.62)	3.97
ASF outbreaks in domestic pigs	Number	0 (0–4)	1.89
ASF outbreaks in wild boar	Number	0 (0–12)	1.37
Number of wild boar per hunting farm	head/hunting farm	15.1 (0–74.5)	1.39
Number of backyards	unit	66.5 (8–435)	1.69
Domestic pig density	Head/km^2^	0.495 (0.07–137.8)	1.38
Population density	Pers/km^2^	14.3 (4.5–3,051.1)	1.31
Percentage of rural population	%	0.445 (0.01–1)	1.48
Wild boar population density	Head/km^2^	0.016 (0.008–0.87)	1.25

**Table 2:** Characteristics of clusters of ASF cases (Bernoulli model) and wild boar population density (normal model), 2011–2022.

Model	Number of cluster (corresponds to the map in Figure 3)	Cluster radius (km)	Start date	End date	ODE/LLR	*p*-Value
Clusters of ASF cases in wild boar (bernoulli model)	1	26.66	01.01.2019	31.12.2019	72.37/145.84	<0.001
	**2**	**48.92**	**01.01.2016**	**31.12.2016**	**10.02/35.33**	<**0.001**
	3	74.86	01.01.2020	31.12.2020	5.52/13.34	<0.001

Wild boar density clusters (normal model)	**4**	**107.41**	**01.01.2016**	**31.12.2016**	**68.02**	**0.001**

*Note*: ODE-observed/expected (the ratio of the observed number of ASF cases to the expected number within a cluster, provided the distribution corresponds to the null hypothesis). LLR (log-likelihood ratio) is a parameter of a statistical test used to test limitations on the parameters of statistical models estimated based on sample rates. Clusters highlighted in bold coincide with space and time.

**Table 3:** Statistical metrics of the models used in the selection of the most effective regression models for determining ASF risk factors in the population of domestic pigs and wild boar.

Parameters	Regression model for ASF in domestic pigs		Regression model for ASF in wild boar	
	Logistic model	Negative binomial model	Logistic model	Negative binomial model
*R* ^2^	**0.70**	0.59	0.22	**0.37**
The Hosmer–Lemeshow test, *p*-value	**0.789**	–	0.602	–
Shapiro–Wilk test, *p*-value	–	0.00006	–	**0.006**
Local moran's I, *z*-score (*p*-value)	**0.23 (0.82)**	2.55 (0.01)	1.78 (0.08)	**0.99 (0.32)**

*Note:* statistically significant model results are highlighted in bold.

**Table 4:** Results of the negative binomial regression model of the number of ASF outbreaks in wild boar in the Nizhny Novgorod region, 2016–2022.

Variable	Estimate	Standard error	*p*-Value
Intercept	−1.659	0.523	0.001
Percentage of water bodies (%)	−0.231	0.099	0.019
Number of hunting farms	0.266	0.075	0.0003
ASF outbreaks in domestic pigs	0.520	0.274	0.057

**Table 5:** Results of the logistic regression model of the presence of ASF outbreaks in domestic pigs in the Nizhny Novgorod region, 2011–2022.

Variables	OR	OR 95 (%) CI	*p*-Value
Intercept	0.439	0.087–2.212	0.318
Number of backyards	1.397	1.003–2.500	0.025
Summary road length	0.997	0.991–1.001	0.053

*Note*: OR; odds ratio, OR 95% CI–boundaries of odds ratio 95 (%) confidence interval.

**Table 6:** The results of the regression analysis of the correlation between the supply of live pigs and pig products with the presence of outbreaks of ASF in domestic and wild pigs in the Nizhny Novgorod region, 2011–2022.

Variables	Import of pork products		Import of live animals	
	Logistic model	Negative Binomial model	Logistic model	Negative binomial model
ASF outbreaks in domestic pigs	**+/0.028**	**+/0.001**	−/0.254	−/0.051
ASF outbreaks in wild boar	+/0.183	**+/0.017**	−/0.648	−/0.685

*Note:* the +/− sign indicates a positive/negative dependence, respectively. The coefficient after the sign indicates the statistical significance criterion *p*-value. Statistically significant results are highlighted in bold.

## Data Availability

Data on ASF outbreaks' locations, and explanatory variables were retrieved from the open sources. The data on wild boar population number can be obtained from the corresponding author per reasonable request.

## Supplementary Materials

Supplementary Materials The supplementary material include maps illustrating the distribution of geospatial variables used in our analysis: Figure S1: wild boar population density in the Nizhny Novgorod oblast by district yearly, 2016–2022. Figure S2: distribution of environmental variables in the Nizhny Novgorod oblast by district. Figure S3: distribution of sociodemographic variables in the Nizhny Novgorod oblast by district. Figure S4: distribution of domestic pig population density and a number of wild boar per hunting farm in the Nizhny Novgorod oblast by district.
