# Supplementary material for: Investigation of Risk Factors Associated with the African Swine Fever Outbreaks in the Nizhny Novgorod Region of Russia, 2011–2022
Source: Transbound Emerg Dis. 2023 Sep 21;2023:6334935. doi: 10.1155/2023/6334935 (PMC12016854; doi:10.1155/2023/6334935)
Supplement: Supplementary Materials — The supplementary material include maps illustrating the distribution of geospatial variables used in our analysis: Figure S1: wild boar population density in the Nizhny Novgorod oblast by district yearly, 2016–2022. Figure S2: distribution of environmental variables in the Nizhny Novgorod oblast by district. Figure S3: distribution of sociodemographic variables in the Nizhny Novgorod oblast by district. Figure S4: distribution of domestic pig population density and a number of wild boar per hunting farm in the Nizhny Novgorod oblast by district. [file 6334935.f1.docx]

**Supplementary material**


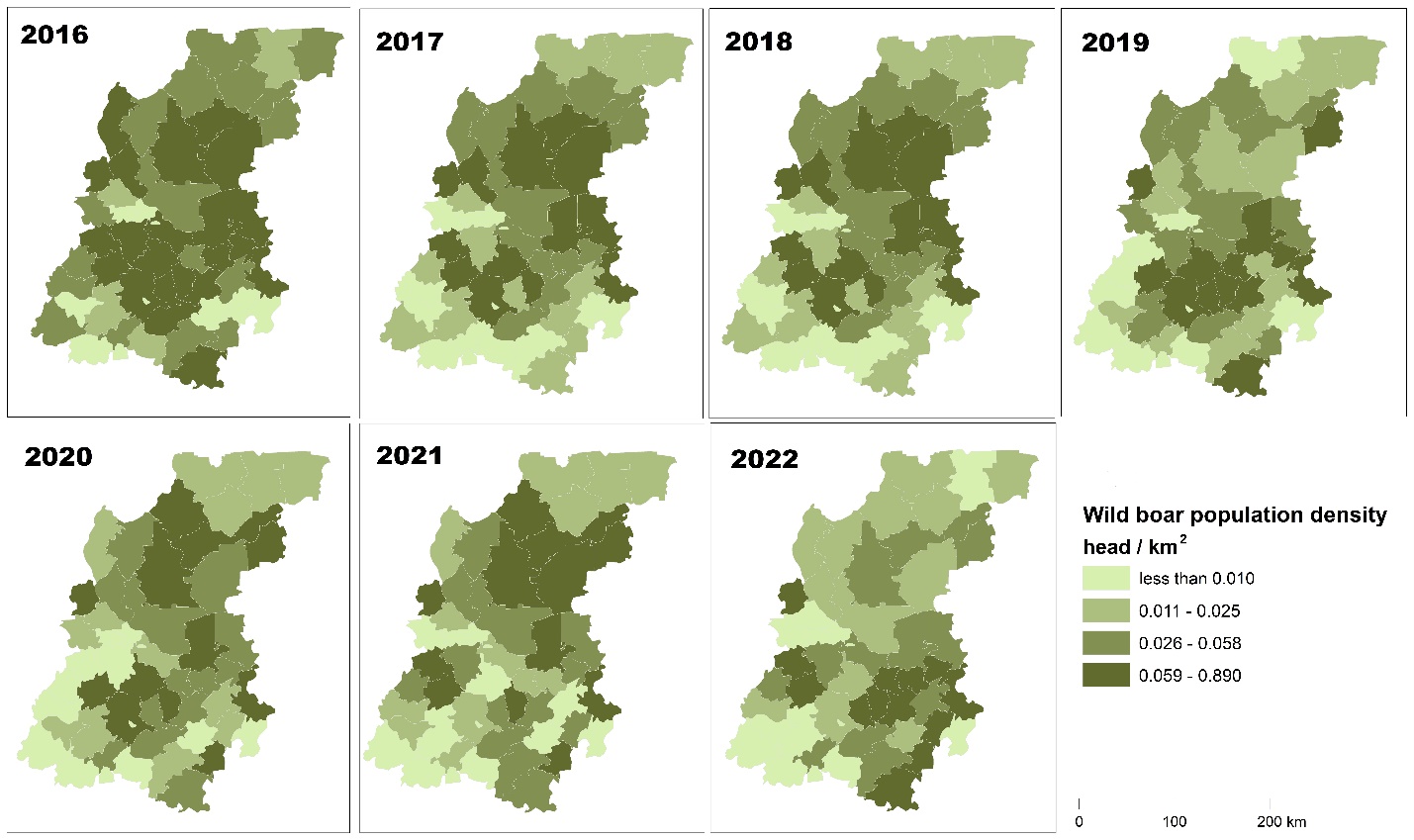


**Figure S1**. Wild boar population density in the Nizhny Novgorod oblast by district yearly, 2016 – 2022


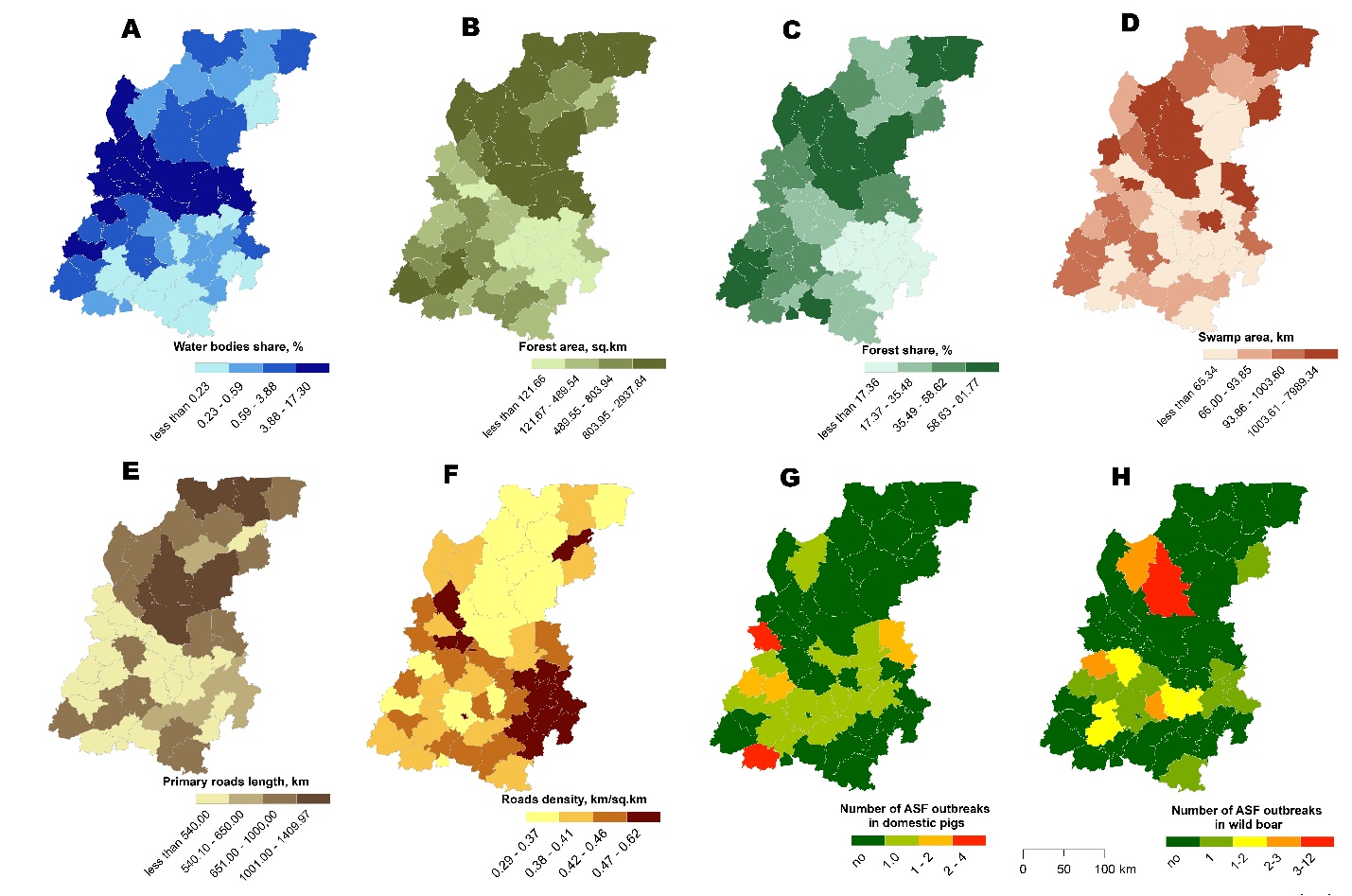


**Figure S2.** Distribution of environmental variables in the Nizhny Novgorod oblast by district


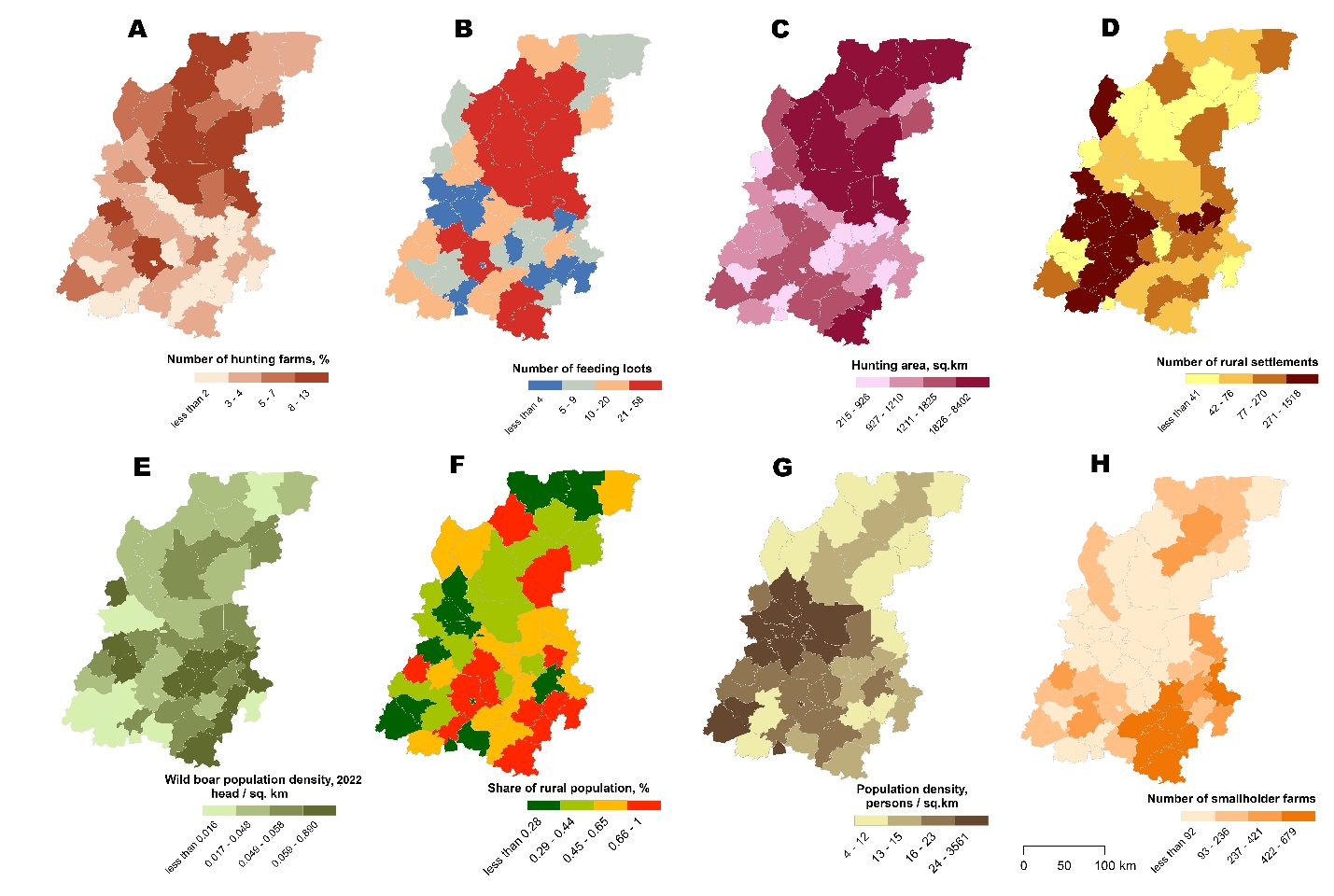


**Figure S3.** Distribution of socio-demographic variables in the Nizhny Novgorod oblast by district

**
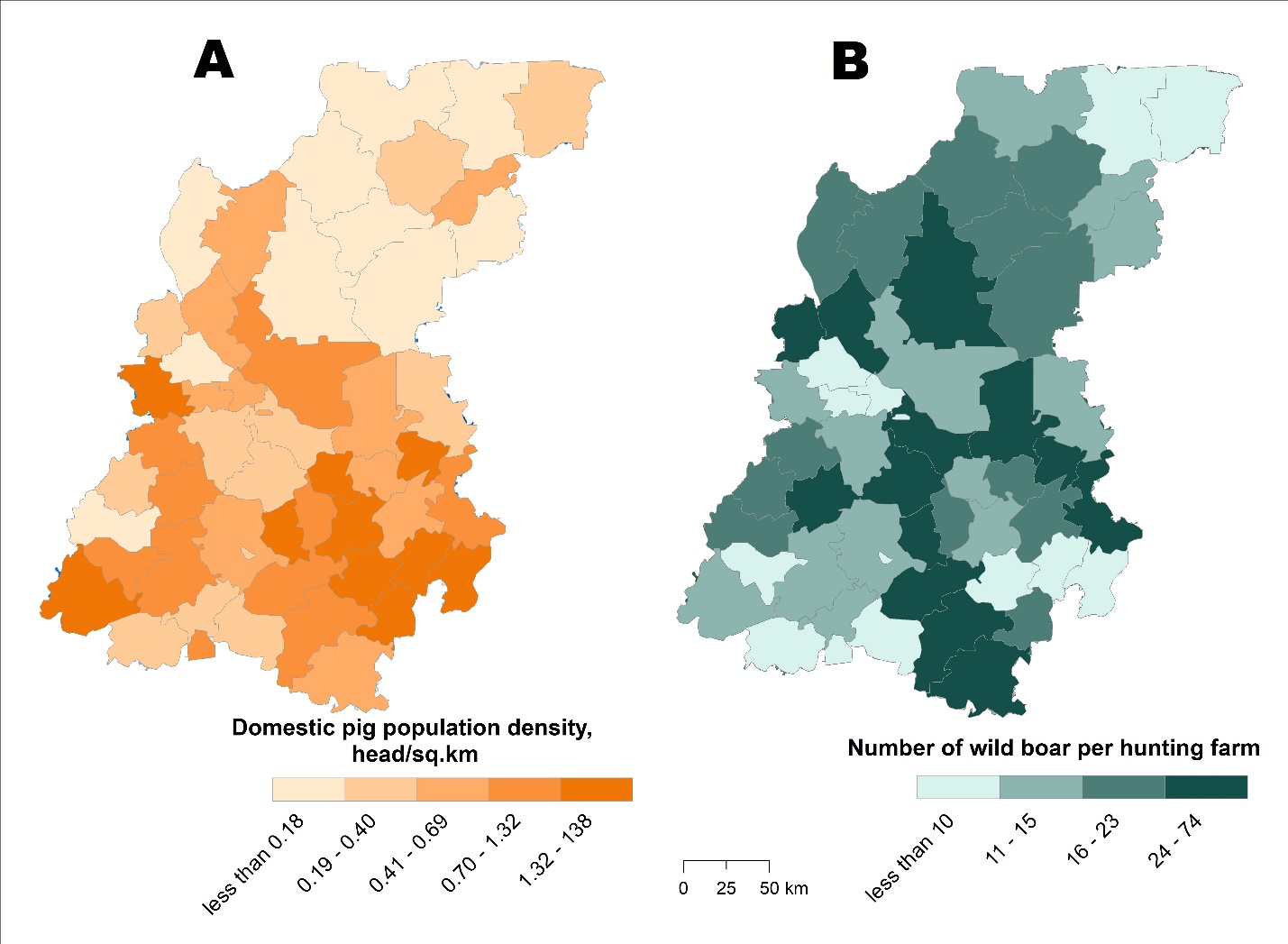
**

**Figure S4.** Distribution of domestic pig population density and a number of wild boar per hunting farm in the Nizhny Novgorod oblast by district
